# Psychotic symptoms in older people without dementia from a Brazilian community-based sample: A seven years’ follow-up

**DOI:** 10.1371/journal.pone.0178471

**Published:** 2017-06-16

**Authors:** Walter Barbalho Soares, Eriton Barros dos Santos, Cássio Machado de Campos Bottino, Helio Elkis

**Affiliations:** 1 Old Age Research Group, Department and Institute of Psychiatry, University of São Paulo Medical School (Proter-Ipq-FMUSP), São Paulo, Brazil; 2 Institute of Mathematics and Statistic, University of São Paulo (IME-USP), São Paulo, Brazil; 3 Department and Institute of Psychiatry, University of São Paulo Medical School (IPq-FMUSP), São Paulo, Brazil; Nathan S Kline Institute, UNITED STATES

## Abstract

**Background:**

Studies of the incidence of psychotic symptoms in elderly people at risk of dementia are scarce. This is a seven year follow up study aiming to determine the incidence of psychotic symptoms and their correlation with other clinical aspects, in particular the rate of development of cognitive impairment.

**Methods:**

Cohort study of a community-based sample of elderly subjects. At study entry in 2004, the sample was composed of 1,125 individuals aged 60 years and older. Of this total, 547 subjects were re-evaluated in 2011 and submitted to the original study protocol. Of these, 199 showed no psychotic symptoms at phase I, while 64 already had psychotic symptoms in 2004.

**Results:**

The incidence of at least one psychotic symptom in the 7 year period was 8.0% (Visual/tactile hallucinations: 4.5%; Persecutory delusions: 3.0%; Auditory hallucinations: 2.5%). Development of psychotic symptoms was associated with epilepsy (OR: 7.75 and 15.83), lower MMSE (OR: 0.72) and reported depression (OR: 6.48). A total of 57.8% of individuals with psychotic symptoms developed cognitive impairment after 7 years. Visual/tactile hallucinations were the only psychotic symptom predictive of this impairment, which was related to lower MMSE and greater functional impairment.

**Conclusions:**

The incidence of psychotic symptoms and the conversion rate to cognitive impairment was in the upper range when compared with previous reports. Visual/tactile hallucinations were the most frequent symptoms and were predictive of cognitive impairment over the 7 year period. A significant relationship was found between the incidence of psychotic symptoms and low MMSE scores, as well as clinical comorbities such as epilepsy, reported depression, diabetes and syphilis.

## Introduction

It is well known that among elderly people with dementia there is a high prevalence of psychotic symptoms. However, studies of psychotic symptoms in the elderly without dementia are scarce. This gap in our understanding may negatively influence the diagnosis and clinical management of this population [1] [2].

Henderson et al. (1998) are among the few investigators that have addressed this question. They found a cumulative incidence of psychotic symptoms of 4.8% in a 3-year follow-up period of a sample of cognitively intact people aged 70 and over [2]. Other authors have estimated the cumulative incidence to be 4.8% in people aged 70–90 based on psychiatric examinations and medical records, 8.0% when key-informant reports were included, and 19.8% among those re-evaluated at 85 years of age [3].

The presence of psychotic symptoms in this population may be of important prognostic value, since it has been shown that cognitively intact individuals with psychotic symptoms have an increased risk of developing dementia (OR: 2,5–3,5) [3,4,5]. Amongst individuals without dementia who exhibited psychotic symptoms, 44% developed dementia during a 20 year follow-up period, with hallucinations (particularly visual hallucinations) being the main symptom associated with more rapid cognitive decline [3] [5].

Our data form part of a large epidemiological study of a cohort of individuals over the age of 60 and living in the city of São Paulo, Brazil. Data from this study (phase I of the present study) have already been published estimating the prevalence of dementia [6] and psychotic symptoms in those without dementia [7] in this Brazilian population.

We previously showed that the prevalence of general psychotic symptoms in this community sample was 9.1%; the breakdown of which was 7.5% auditory hallucinations, 7.8% other types of hallucinations and 2.9% persecutory delusions. A significant relationship was found between the presence of psychotic symptoms and a lower Mini Mental State Examination score, fewer years of schooling as well as a lower socioeconomic status. Comorbidities were also very frequent such as head trauma, depression, diabetes, Chagas Disease, arthritis and alcohol abuse [7].

This same cohort was followed up over seven years and the results of this period of observation are the focus of the present paper. Here we examine the incidence of psychotic symptoms in subjects over 60 years of age without dementia, the rate of development of cognitive impairment and the correlation of these symptoms with socioeconomic and clinical characteristics.

## Methods

### Sample selection

This study examines a sub-population of a larger clinical-epidemiological study that investigated the prevalence and etiology of dementia in a community-based sample of individuals aged over 60 and living in São Paulo, Brazil [6]. We selected those subjects that, at enrolment, did not have cognitive impairment or clinically significant Depressive Symptoms (CSDS).

The study protocol was approved by the University of São Paulo General Hospital (CAPPesq—Protocol number: 1113/07), and all the subjects gave their informed written consent.

According to census data from 2000, the city of São Paulo had approximately 970,000 people who were over 60 years of age [8]. This census ranked 96 city districts from the wealthiest to the poorest, which were divided into 3 groups; one district of each socioeconomic stratum was chosen to represent the upper, middle and lower groups [6].

As previously described, the dementia prevalence of 7% was based on a published study of a Brazilian elderly population [9]. Using the same parameters, we calculated the required sample size to be 1,100 (using the Epi Info 6 software—https://www.cdc.gov/epiinfo/pc.html). This number was then multiplied by 1.5 as a correction factor and, adding 20% for the possible losses, we obtained a final sample size of 2,062 individuals [6].

Together, the three chosen districts had an elderly population of 64,760. Thirty areas from each district were selected, and 10 houses were randomly chosen from each area. After local media advertisement, the researchers visited 8,042 houses. 2,233 individuals older than 60 years were selected and from these 1,563 agreed to participate. They were asked to respond to a questionnaire about their socioeconomic and clinical status. They were also evaluated using a number of instruments: the Mini Mental State Examination (MMSE) [10], the Fuld Object Memory Evaluation (FOME) [11], the Informant Questionnaire on Cognitive Decline in the Elderly (IQCODE) [12], the Bayer Activities of Daily Living scale (B-ADL) [13] and the Scale to Screen Depressive Symptoms in Older People (D-10) [14]). These 1,563 individuals constituted the sample of the large clinical-epidemiological study about dementia and cognitive impairment [7] of which our study is part.

Individuals were considered positive in the screen for dementia or cognitive and functional impairment if they scored below the cutoff point on one of the cognitive tests (MMSE < 20—illiterate subjects; MMSE < 25–1–4 years of schooling; MMSE < 27–5–8 years of schooling; MMSE < 28 –more than 9 years of schooling; FOME < 35) and above the cutoff point on one functional scale (IQCODE > 3.4 and B-ADL > 3.19) [7]. The cutoff point for clinically significant Depressive Symptoms (CSDS) on the D-10 scale was ≥ 7 [15].

From the initial sample of 1,563 subjects, we excluded individuals who screened positive for dementia, cognitive and functional impairment, or CSDS, resulting in 1,125 individuals, which was the sample of the first phase of our study (2004), which has been reported elsewhere [7].

In December 2010, we contacted the subjects from the first phase sample using letters, e-mails and phone calls to explain and invite them to the second phase. After one month (January 2011), 690 new interviews were conducted, which included the reapplication of the questionnaire from the first phase.

**Fig 1:**
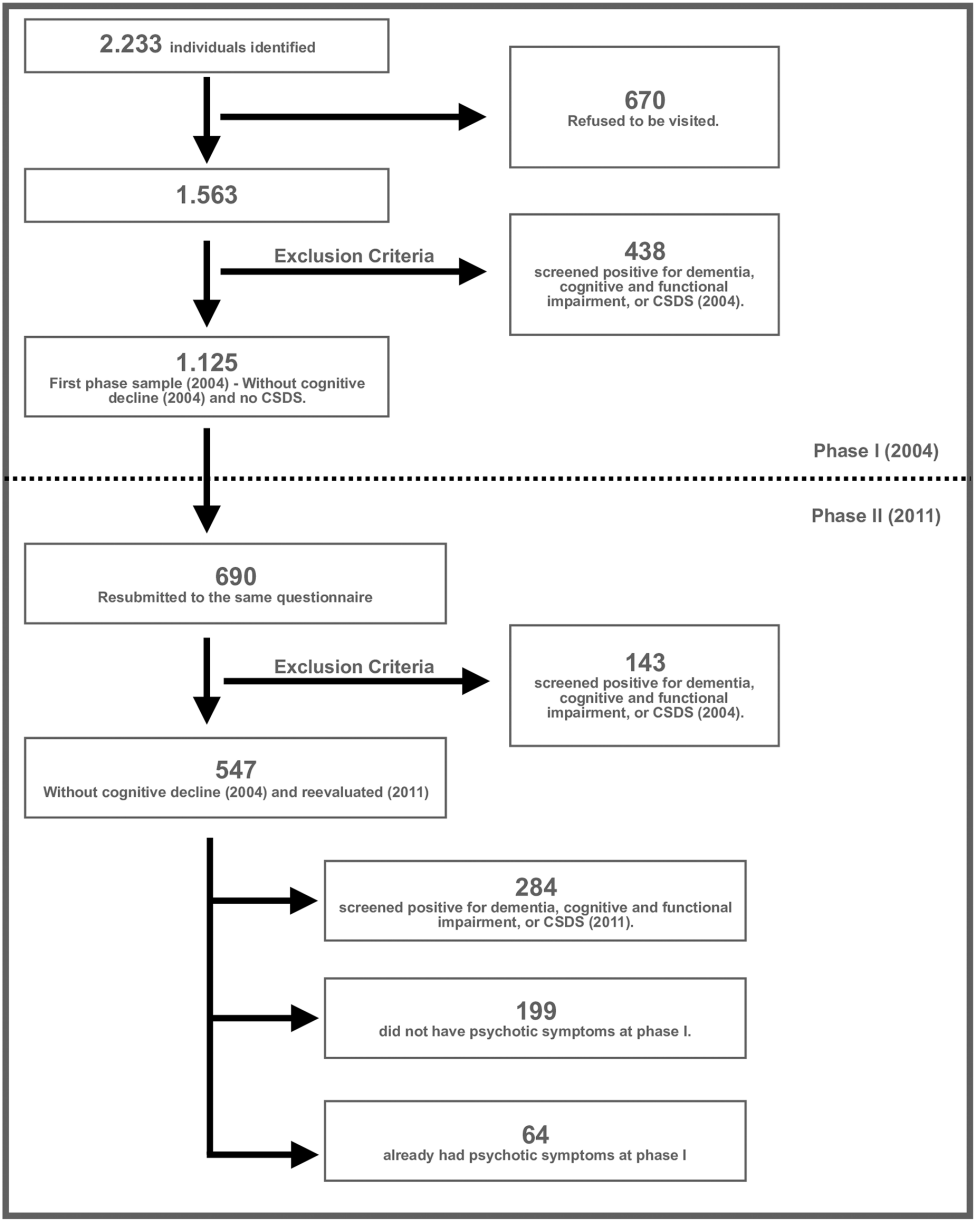
Study sample flowchart.

From this sample of 690 individuals, 547 with no initial cognitive decline (2004) were re-evaluated using the same protocol as the original study 7 years earlier. From this sample, 284 had a positive screening for dementia or CSDS in 2011. Of the remaining 263 subjects, 199 did not have psychotic symptoms at phase I and thus constituted the sample used to evaluate the incidence of psychotic symptoms, while the 64 individuals who already had psychotic symptoms in 2004 constituted the sample used to evaluate rate of conversion to cognitive decline. (Fig 1). (see Fig 1)

### Instruments

A questionnaire was used to screen for dementia, cognitive and functional impairment and CSDS. It contained questions regarding clinical and socioeconomic status. It was divided into the following sections: subject personal information, caregiver personal information (the caregiver being required to be a family member or a close friend) and personal history and habits. Trained interviewers investigated the presence of previous medical diagnoses. Common medical comorbidities such as hypertension, diabetes and cerebral vascular accident were evaluated through objective and direct questions. Family history was also explored. Subjects were questioned directly when cognitively intact and a collateral history was obtained when they were cognitively impaired. Issues around the use of health services were explored, including the use of public services via the universal public health system available by right to all Brazilians, or private healthcare such as health insurance or private consultations, or no use of health services, either in terms of primary care, secondary care (inpatient or outpatient) or tertiary centres.

Other aspects were also evaluated such as instrumental and physical activities, depressive symptoms (D-10 [14]), socioeconomic status based on the ABIPEME (Brazilian Association of Market Research) questionnaire, as well as cognitive/functional evaluation using the MMSE [10], FOME [11], IQCODE [12] and B-ADL [13]–last both completed by the caregiver.

We used the following three questions for the screening of psychotic symptoms, which were extracted from the Cambridge Mental Disorders of the Elderly Examination (CAMDEX), adapted for and translated to Brazilian Portuguese [16,17]: Do you have, or have you ever had, the experience of hearing things that other people do not? Do you ever have the experience of seeing things that other people do not? Do you ever believe that people are watching you, spying on you, or plotting against you?

### Statistical analysis

The data were stored and analyzed using the Statistical Package for the Social Sciences version 16.0 for Windows (SPSS, developed by SPSS Inc. in Chicago, USA). Initially, we calculated the incidence of all psychotic symptoms in 7 years, then the incidence for each category.

The Mann–Whitney test was used to compare continuous variables between groups of individuals with and without psychotic symptoms. The same test was used to compare groups of cognitively impaired and cognitively intact individuals with psychotic symptoms. The Kruskall- Wallis test was used to compare samples of individuals with no psychotic symptoms against those with at least one, two or three psychotic symptoms, respectively. The chi-squared test was used to compare categorical variables within the same groups.

To evaluate the association between important variables and possible risk factors for psychotic symptom development, a stepwise backward multiple logistic regression was calculated for each of the psychotic symptoms (dependent variables) as well as the following variables that reached statistical significance at the bivariate analysis: MMSE, diabetes, epilepsy, reported depression and syphilis. Variables that had significance in the first phase of the study such as gender, years of schooling, B-ADL socioeconomic classification and Chagas Disease were entered as predictors or independents variables in the second phase.

Another stepwise backward multiple logistic regression was calculated to verify an association between each psychotic symptom (independent variables) with the development cognitive impairment (dependent variable) in the sample of 64 individuals who already had psychotic symptoms at 2004. The level of significance adopted was 5%.

## Results

### Sample description

We studied two sub-samples, one without and the other with psychotic symptoms in Phase I. In both sub-samples, more than 90% of the participants were between 80 and 90 years of age, and were predominantly women (almost 70%). More than 80% of the participants were married or widowed. Both sub-samples had 4 important characteristics: participants were retired or had no labor activity (80.9 and 82.8%), had some type of clinical comorbidity (83.4 and 87.5%), belonged to the B or C socioeconomic classes (75.3 and 76.6%) and were not using psychotropic medication (82.9 and 71.9%) (Table 1).

**Table 1:** Demographic characteristics of the sample.

Variables		Individuals *N* (%)	
		Without psychotic symptoms in phase I	With psychotic symptoms in phase I
Age group	80–90 years	198 (99.5)	60 (93.8)
	More than 90 years	1 (0.5)	4 (6.3)
Gender	Male	72 (36.2)	17 (26.6)
	Female	127 (63.8)	47 (73.4)
Civil Status	Single	17 (8.5)	2 (3.1)
	Married	99 (49.7)	25 (39.1)
	Divorced	6 (3.0)	6 (9.4)
	Widowed	73 (36.7)	31 (48.4)
	Other	2 (1.0)	0 (0.0)
Years of Schooling	0	0 (0.0)	1 (2.1)
	1 a 4	71 (38.0)	27 (56.3)
	5 a 8	27 (14.4)	5 (10.4)
	9 a 12	27 (14.4)	6 (12.5)
	13 or more	62 (33.2)	9 (18.8)
Socioeconomic Classification	A	41 (20.6)	6 (9.4)
	B	93 (46.7)	25 (39.1)
	C	57 (28.6)	24 (37.5)
	D	8 (4.0)	9 (14.1)
	E	0 (0.0)	0 (0.0)
Labour Activity	Yes	16 (8.0)	3 (4.7)
	No	161 (80.9)	53 (82.8)
Comorbidity	Yes	166 (83.4)	56 (87.5)
	No	33 (16.6)	8 (12.5)
Psychotropic Medication Use	Yes	34 (17.1)	17 (26.6)
	No	165 (82.9)	46 (71.9)

### Psychotic symptoms incidence

The incidence of at least one psychotic symptom over the 7 year follow up was 8.0%, and the incidence of at least two psychotic symptoms was 1.0%. Visual and tactile hallucinations were the most frequent (4.5%), followed by persecutory delusions (3.0%) then auditory hallucinations (2.5%).

### Cognitive aspects

The individuals who developed psychotic symptoms had a lower mean MMSE score at Phase II than subjects without psychotic symptoms (22.8 vs. 25.9, p ≤ 0.01). Individuals who reported to have persecutory delusions had a particularly low MMSE mean score (19.0). Between groups there was no statistical difference in terms of activities of daily living (B-ADL) or years of schooling (Tables 2 and 3).

**Table 2:** Relationship between type of psychotic symptom, cognitive impairment (MMSE), functional status (B-ADL) and use of health services.

	MMSE	B-ADL	Health Service Used			
			No	Private	Insurance	Public
With auditory hallucinations	24.00^b^	2.25^b^	0.0^b^	0.0^b^	0.0^b^	100,0^b^
Without auditory hallucinations	25.69^b^	1.81^b^	0.5^b^	22.0^b^	44.0^b^	33.0^b^
With visual/tactile hallucinations	24.11^b^	2.28^b^	0.0^b^	11.1^b^	44.4^b^	44.4^b^
Without visual/tactile hallucinations	25.72^b^	1.80^b^	0.5^b^	21.9^b^	42.8^b^	34.2^b^
With persecutory delusions	19.00^a^	2.30^b^	0.0^b^	0.0^b^	50.0^b^	50.0^b^
Without persecutory delusions	25.84^a^	1.80^b^	0.5^b^	22.2^b^	42.3^b^	34.4^b^
No psychotic symptoms	25.94^a^	1.77^b^	0.6^b^	23.0^b^	43.8^b^	32.0^b^
1 psychotic symptom	22.81^a^	2.26^b^	0.0^b^	6.2^b^	31.2^b^	62.5^b^
2 psychotic symptoms	21.50^a^	2.38^b^	0.0^b^	0.0^b^	50.0^b^	50.0^b^

^a^ p ≤ 0.01

^b^ p > 0.05

**Table 3:** Relation between psychotic symptoms, years of schooling and socioeconomic status.

	Years of schooling (%)			Socioeconomic classes (%)				
	1–4	5–8	9–11	A	B	C	D	E
With auditory hallucinations	50.0 ^b^	25.0 ^b^	25.0 ^b^	0.0^b^	40.0^b^	40.0^b^	20.0^b^	0.0^b^
Without auditory hallucinations	37.7 ^b^	14.2 ^b^	48.1 ^b^	21.1^b^	46.9^b^	28.4^b^	3.6^b^	0.0^b^
With visual/tactile hallucinations	55.6 ^b^	11.1 ^b^	33.3 ^b^	11.1^b^	33.3^b^	55.6^b^	0.0^b^	0.0^b^
Without visual/tactile hallucinations	37.1 ^b^	14.6 ^b^	48.3 ^b^	21.1^b^	47.4^b^	27.4^b^	4.2^b^	0.0^b^
With persecutory delusions	75.0 ^b^	0.0 ^b^	25.0 ^b^	0.0^b^	33.3^b^	50.0^b^	16.7^b^	0.0^b^
Without persecutory delusions	36.8 ^b^	14.8 ^b^	48.4 ^b^	21.4^b^	47.4^b^	27.6^b^	3.6^b^	0.0^b^
No psychotic symptoms	36.0 ^b^	14.5 ^b^	49.4 ^b^	22.1^b^	48.1^b^	26.5^b^	3.3^b^	0.0^b^
1 psychotic symptom	11.3 ^b^	15.4 ^b^	23.1 ^b^	6.2^b^	31.2^b^	50.0^b^	12.5^b^	0.0^b^
2 psychotic symptoms	50.0 ^b^	0.0 ^b^	50.0 ^b^	0.0^b^	50.0^b^	50.0^b^	0.0^b^	0.0^b^

^a^: p ≤ 0.01;

^b^ > 0.05.

### Socioeconomic aspects

Differences in socioeconomic class and use of health services did not reach statistical significance between groups. However, there is a clear trend with the majority of patients with psychotic symptoms belonging to classes C and D and those without belonging to classes A and B. The use of the public health system and health insurance was more frequent among elderly people with psychotic symptoms (Tables 2 and 3).

### Comorbidities

Almost 84% of the sample had at least one comorbidity. A large proportion of individuals with auditory hallucinations (when compared to those without) were shown to have syphilis (20.0% vs. 0.5%, p ≤ 0.01). Diabetes was more frequent in individuals with visual/tactile hallucinations (33.3% vs. 20.5%, p ≤ 0.05). Depression was reported more frequently by those with persecutory delusions compared to those without delusions (50.0% vs. 12.6%, p ≤ 0.05) (Table 4).

**Table 4:** Frequency of comorbidities by psychotic symptom type (%).

	CVA	CET	DEP	DM	EPI	AMI	SYP	CD	ART
With auditory hallucinations	0.0^c^	0.0^c^	20.0^c^	40.0^c^	20.0^c^	20.0^c^	20.0^a^	0.0^c^	60.0^c^
Without auditory hallucinations	3.1^c^	2.6^c^	13.5^c^	20.6^c^	1.6^c^	8.8^c^	0.5^a^	0.5^c^	21.1^c^
With visual/tactile hallucinations	0.0^c^	0.0^c^	0.0^c^	33.3^b^	11.1^c^	0.0^c^	0.0^b^	0.0^c^	0.0^c^
Without visual/tactile hallucinations	0.0^c^	2.6^c^	14.3^c^	20.5^b^	1.6^c^	9.5^c^	1.1^b^	0.5^c^	23.2^c^
With persecutory delusions	0.0^c^	0.0^c^	50.0^b^	16.7^c^	16.7^c^	16.7^c^	0.0^c^	0.0^c^	16.7^c^
Without persecutory delusions	3.1^c^	2.6^c^	12.6^b^	21.4^c^	1.6^c^	8.9^c^	1.0^c^	0.5^c^	22.4^c^
No psychotic symptoms	3.3^c^	2.8^c^	12.8^c^	19.9^b^	1.1^a^	8.8^c^	0.6^a^	0.6^c^	22.1^c^
1 psychotic symptom	0.0^c^	0.0^c^	25.0^c^	37.5^b^	6.2^a^	12.5^c^	6.2^a^	0.0^c^	25.0^c^
2 psychotics symptoms	0.0^c^	0.0^c^	0.0^c^	0.0^b^	50.0^a^	0.0^c^	0.0^a^	0.0^c^	0.0^c^

^a^: p ≤ 0.01;

^b^: p ≤ 0.05;

^c^: p > 0.05;

CVA: cerebrovascular accident; CET: Head Trauma; DEP: Reported Depression; DM: Diabetes Mellitus; EPI: Epilepsy; AMI: Acute Myocardial Infarction; SYP: Syphilis; CD: Chagas Disease; ART: Arthritis.

When we compared individuals who reported at least one psychotic symptom with those without psychotic symptoms, there was a higher frequency of epilepsy (6.2% vs. 1.1%, p ≤ 0.01), diabetes (37.5% vs. 19.9%, p ≤ 0.05) and syphilis (6.2% vs. 0.6%, p ≤ 0.01). In those elderly people who reported at least 2 psychotic symptoms epilepsy was again more frequently observed (50.0% vs. 1.1%, p ≤ 0.01) (Table 4).

### Logistic regression

At phase II, Epilepsy was shown to be a statistically predictive variable for auditory (OR: 15.83) and visual or tactile hallucinations (OR: 7.75), while lower MMSE scores as well as reported depression were predictive of persecutory delusions (OR: 6.48) (Table 5).

**Table 5:** Different types of psychotic symptoms and respective predictors.

	Beta (SE)	OR	95% IC Exp (B)	p
*Auditory Hallucinations*				
Epilepsy	2.76 (1.26)	15.83	1.34–187.25	0.028
*Visual/Tactile Hallucinations*				
Epilepsy	2.05 (1.21)	7.75	0.72–83.00	0.091
*Persecutory Delusions*				
MMSE	- 0.32 (0.09)	0.72	0.60–0.88	0.00
Depression	1.87 (0.97)	6.48	0.97–43.47	0.05

### Conversion to cognitive impairment

In the present study, 57.8% of individuals with psychotic symptoms and without dementia at Phase I developed cognitive impairment during the 7 year follow up period. Comparison of these individuals with those who did not develop cognitive impairment showed that lower MMSE mean score (19.5 vs. 24.0, p ≤ 0.01) and greater functional impairment (B-ADL 3.3 vs. 2.1, p ≤ 0.01) were predictors of conversion. Of the psychotic symptoms studied, the presence of visual/tactile hallucinations was the only statistically predictive variable for subsequent development of cognitive impairment (OR: 5.66) (Table 6).

**Table 6:** Psychotic symptoms and their associations to cognitive impairment conversion rates, after 7 years follow-up.

	Beta (SE)	OR	95% IC Exp (B)	p
Auditory Hallucinations	1.77 (1.17)	5.86	0.58–58,74	0.132
Visual/Tactile Hallucinations	1.73 (0.87)	5.66	1.04–30.86	0.045
Persecutory Delusion	- 24.41 (30468.61)	2.48	0.00 –a	0.999

## Discussion

Although there are two previous studies [2,3], to the best of our knowledge this is the first study to have evaluated the incidence of psychotic symptoms in the elderly population of a developing country. The main finding of this study is an incidence of 8% of at least one psychotic symptom in a seven-year follow-up period, which is higher than the 4.8% incidence previously reported in the literature [2,3].

Although these authors also argued that using information provided by key informants, the incidence could be as high as 8.0% [3], the present study specified more clearly the incidence of each type of psychotic symptom and used a broader cognitive impairment screening tool. However, we have found a similar incidence rate of visual or tactile hallucinations (4.5%) and persecutory delusions (3%) as previously reported elsewhere [3]. In the first phase of our study, similar to the second phase, visual/tactile hallucinations were found to be the most prevalent (7.8%) followed by auditory hallucinations (7.5%) then persecutory delusions (2.9%) [7].

### Cognitive performance

Individuals with at least one psychotic symptom had an average of 3 points lower MMSE score when compared with those without such symptoms. Indeed Henderson et al., 1998 have already reported an association between lower MMSE scores and the incidence of psychotic symptoms in older subjects without cognitive impairment [2]. In the first phase of our study, individuals with persecutory delusions had a lower MMSE score than those without persecutory delusions (more than 3 points), which we again observed in the second phase, but with a larger difference (more than 6 points) [7]. This may suggest that it is the presence of persecutory delusions in particular that has the greatest impact on cognition, more so than hallucinations.

Although not statistically significant, a higher B-ADL score was found in subjects with psychotic symptoms. B-ADL scores from the second phase were higher than those from the first study phase, which indicates a possible functional decline during the 7 years between evaluations.

### Socioeconomic aspects

This study was the first to examine the correlation between psychotic symptoms and socioeconomic variables. We found a trend for an association between the presence of psychotic symptoms and lower socioeconomic status; psychotic symptoms occurred more frequently in classes C and D than in classes A and B. This is a similar finding to the first study phase, where the association between psychotic symptoms and lower socioeconomic class was evident [7].

We also observed a greater tendency to use the public health system and health insurance amongst individuals with psychotic symptoms, although not statistically significant. This knowledge could improve the organization of these services and help to provide better diagnostic screening and treatment of such patients.

### Comorbidities

A high level of medical comorbidity was encountered in this sample, with more than 83% of subjects reporting at least one condition. Older populations have a vulnerability for the co-occurrence of physical and mental health problems [2], a relationship that has been well described in the literature since the first studies of psychotic symptoms in elderly people [2,18].

Epilepsy, depression, syphilis and diabetes were the most strongly associated with psychotic symptoms. Epilepsy was significantly more frequently reported by both individuals with at least one and at least two psychotic symptoms, and 2–7% of patients suffering from epilepsy had psychotic symptoms [19]. This association has several possible causes: subictal limbic system activity, use of anticonvulsants, changes in dopaminergic receptors, changes in cerebral blood flow induced by repeated epileptic crises and autoimmune reactions [19,20,21]. New-onset epilepsy in older people has a strong association with dementia [20], which is frequently associated with psychotic symptoms. Finally, the presence of psychotic symptoms in epileptic individuals may be explained by a possible prodromal dementia state, which has been reported in the literature [20].

The finding of an association between reported depression and persecutory delusions could be related to a possible common origin (degenerative brain changes) [2] or to psychotic depression.

Individuals with auditory hallucinations more frequently reported a history of syphilis when compared with individuals without psychotic symptoms. It is known that neurosyphilis can be the underlying cause of various psychiatric syndromes, such as dementia, cognitive impairment and psychotic disorders [22]. However, the total number of subjects with a history of syphilis was small, and our data were conflicting. Visual/tactile hallucinations were more frequently found in individuals without syphilis, and while a history of syphilis was more frequently encountered in those with at least one psychotic symptom, this did not hold true in those with at least two psychotic symptoms.

### Possible risk factors for psychotic symptoms incidence

Once the first phase of the study was complete [7], we investigated possible risk factors for psychotic symptom incidence using logistic regression. We found that epilepsy was the only predictive factor for hallucinations. Epilepsy increased by almost 16 fold the rate of incident cases of auditory hallucinations and by almost 8 fold that of visual/tactile hallucinations. The possible explanations for this are discussed above.

Importantly we demonstrated a relationship between cognitive impairment and psychotic symptoms. We found that a decrease of one point on the MMSE was associated with a 32% increased risk of development persecutory delusions. The incidence of psychotic symptoms is known to be related to lower scores on the MMSE, the presence of physical symptoms as well as living in a sheltered home [2]. During our first phase, individuals with psychotic symptoms performed worse on the MMSE than those without such symptoms [7]. Lower MMSE scores were associated with auditory hallucinations and persecutory delusions [7]; this association with persecutory delusions was strongly reinforced in this second phase.

Reported depression increased the chances of reporting persecutory delusions by 6.5 times. The association between depression and psychotic symptoms is well established in the literature [2,3,7]. As discussed above, this association may be due to psychotic depression or to a possible common origin.

### Conversion to cognitive impairment

This is the first study to examine psychotic symptoms in elderly individuals without dementia, as well as the conversion rate to cognitive impairment, in the context of a developing country. We found that almost 58% of the non-demented individuals with psychotic symptoms developed cognitive impairment within the 7 years follow-up. This is higher than previously reported figures that range between 18.5–44.0% [3,4].

The presence of psychotic symptoms increases the risk of developing a dementia syndrome by 2.5–3.5 fold [3,4,5]. The cognitive impairment seen in these individuals develops more rapidly than in the general population and occurs especially in non-memory functions [5]. The interval mean between the onset of a psychotic symptom and the development of cognitive impairment is 5 years [3].

It was found that visual/tactile hallucinations increased the chance of developing cognitive impairment by almost 6 fold. A similar association has been reported where by hallucinations, particularly visual hallucinations, as well as persecutory delusions have been shown to be related to an increase in the incidence of cognitive impairment in elderly individuals, although an exact relative risk had not been established [3,4].

The association between dementia and psychotic symptoms is well established [23, 24]. The present study clearly shows that in elderly people without dementia, the presence of psychotic symptoms increases the risk of developing cognitive impairment [3, 4, 5].

### Limitations

Women are over represented in our study sample, as such it is unclear how well this reflects the elderly population of Sao Paulo as a whole.

The results of this study were obtained over a period of 7 years, this resulted in significant loss to follow-up [25]. In fact, 28.6% of the subjects from the first phase of the study could not subsequently be found and 18.8% had died.

Responses to interview questions given the patient or their caregiver may be unreliable and give an under- or overestimation of some variables, this is a limitation of our study as it was heavily reliant on this technique. However, researchers evaluating older subjects found there to be a significant correlation between subject and caregiver reporting of health problems [26].

Finally, the CAMDEX screening tool for psychotic symptoms does not take into account time frame or frequency.

## Conclusion

We found an incidence of psychotic symptoms and a conversion rate to cognitive impairment that was in in the upper range compared to other reports in the literature. Visual or tactile hallucinations had the highest incidence and were predictive of cognitive impairment. Important relationships were found between the incidence of psychotic symptom and MMSE score, epilepsy, depression, diabetes and syphilis.

Future studies are warranted to better our understanding, and to help prevent the development of psychosis and cognitive impairment in the elderly population.

## Supporting information

S1 File Data bank. (SAV)

## References

[1] Sigtrom R, Skoog I, Sacuiu S, Karlsson B, Klenfeldt IF, Waern M, et al: The prevalence of psychotic symptoms and paranoid ideation in non-demented population samples aged 70–82 years. Int J Geriatr Psychiatry 2009; 24:1413–9. doi: 10.1002/gps.2278 19347837 10.1002/gps.2278

[2] Henderson AS, Korten AE, Levings C, Jorm AF, Christensen H, Jacomb PA, et al Psychotic symptoms in the elderly: a prospective study in a population sample. Int J Geriatr Psychiatry 1998; 13:484–92. 9695039 10.1002/(sici)1099-1166(199807)13:7<484::aid-gps808>3.0.co;2-7

[3] Ostling S, Palsson SP, Skoog I. The incidence of first-onset psychotic symptoms and paranoid ideation in a representative population sample followed from age 70–90 years. Relation to mortality and later development of dementia. Int J Geriatr Psychiatry 2007; 22:520–8. doi: 10.1002/gps.1696 17117394 10.1002/gps.1696

[4] Ostling S, Skoog I. Psychotic symptoms and paranoid ideation in a nondemented population-based sample of the very old. Arch Gen Psychiatry 2002; 59:53–9. 11779282 10.1001/archpsyc.59.1.53

[5] Kohler S, Allardyce J, Verhey FRJ, McKeith IG, Matthews F, Brayne C, Savva GM. Cognitive Decline and Dementia Risk in Older Adults With Psychotic Symptoms: A Prospective Cohort Study. Am J Geriatr Psychiatry 2013; 21:119–128. doi: 10.1016/j.jagp.2012.10.010 23343485 10.1016/j.jagp.2012.10.010

[6] Bottino CM, Azevedo D Jr, Tatsch M, Hototian SR, Moscoso MA, Folquitto J, et al Estimate of dementia prevalence in a community sample from São Paulo, Brazil. Dement Geriatr Cogn Disord 2008; 26:291–9. doi: 10.1159/000161053 18843181 10.1159/000161053

[7] Soares WB, Ribeiz SRI, Bassitt DP, De Oliveira MC, Bottino CMC. Psychotic symptoms in older people without dementia from a Brazilian community-based sample. Int J Geriatr Psychiatry 2015; 30(5):437–445. doi: 10.1002/gps.4156 24990116 10.1002/gps.4156

[8] Instituto Brasileiro de Geografia e Estatística. Perfil dos Idosos Responsáveis pelos Domicílios no Brasil 2000. Instituto Brasileiro de Geografia e Estatística, Rio de Janeiro, 2002.

[9] Herrera E Jr, Caramelli P, Silveira AS, Nitrini R. Epidemiologic survey of dementia in a community-dwelling Brazilian population. Alzheimer Dis Assoc Disord 2002; 16: 103–8. 12040305 10.1097/00002093-200204000-00007

[10] Folstein MF, Folstein SE, Mchugh PR. Mini mental state: a practical method for grading the cognitive state of patients for the clinician. *J* Psychiatr Res 1975; 12:189–98. 1202204 10.1016/0022-3956(75)90026-6

[11] Fuld PA, Masur DM, Blau AD, Crystal H, Aronson MK. Object-memory evaluation for prospective detection of dementia in normal functioning elderly: predictive and normative data. J Clin Exp Neuropsychol 1990; 12:520–8. doi: 10.1080/01688639008400998 2211974 10.1080/01688639008400998

[12] Jorm AF, Jacomb PA. The Informant Questionnaire on Cognitive Decline in the Elderly (IQCODE): socio-demographic correlates, reliability, validity and some norms. Psychol Med 1989; 19:1015–22. 2594878 10.1017/s0033291700005742

[13] Lehfeld H, Reisberg B, Finkel S, Kanowski S, Wied V, Pittas J, et al Informant-rated activities-of-daily-living (ADL) assessments: results of a study of 141 items in the U.S.A., Germany, Russia, and Greece from the International ADL Scale Development Project. Alzheimer Dis Assoc Disord 1997; 11(suppl 4):39–44. 9339272

[14] Barczak DS. Validação de Escala para Rastreamento de Depressão em Idosos: Importância de um Teste de Aplicação Rápida. Masters Dissertation, Department of Medicine, University of São Paulo, 2011. http://www.teses.usp.br/teses/disponiveis/5/5142/tde-19032012-/

[15] Barcelos-Ferreira R, Pinto JA Jr, Nakano EY, Steffens DC, Litvoc J, Bottino CM. Clinically significant reportede depressionand associated factors in community elderly subjects from Sao Paulo, Brazil. Am J Geriatr Psychiatry 2009; 17:582–90. 19546654 10.1097/JGP.0b013e3181a76ddc

[16] Roth M, Tym E, Mountjoy CQ, Huppert FA, Hendrie H, Verma S, et al CAMDEX A standardized instrument for the diagnosis of mental disorder in the elderly with special reference to the early detection of dementia. Br J Psychiatry 1986; 149:698–709. 3790869 10.1192/bjp.149.6.698

[17] Sampaio SG, Lourenço RA. O CAMDEX-R e o Diagnóstico de Demência no Brasil—Tradução e Adaptação Transcultural de Entrevista com Acompanhante. Acta Med Port 2009; 22:571–8. 19944041

[18] Christenson R, Blazer D. Epidemiology of persecutory ideation in an elderly population in the community. Am J Psychiatry 1984; 141:1088–91. doi: 10.1176/ajp.141.9.1088 6235752 10.1176/ajp.141.9.1088

[19] Clancy MJ, Clarke MC, Connor DJ, Cannon M, Cotter DR. The prevalence of psychosis in epilepsy: a systematic review and meta-analysis. BMC Psychiatry. 2014; 14:75 doi: 10.1186/1471-244X-14-75 24625201 10.1186/1471-244X-14-75 PMC3995617

[20] Martin RC, Faught E, Richman J, Funkhouser E, Kim Y, Clements K, et al Psychiatric and neurologic risk factors for incident cases of new-onset epilepsy in older adults: Data from U.S. Medicare beneficiaries. 2014; 55(7):1120–7. 10.1111/epi.12649 24902475

[21] Pollak TA, Nicholson TR, Mellers JDC, Vicent A, David AS. Epilepsy-related psychosis: A role for autoimmunity? Epilepsy & Behaviour. 2014; 36:33–38. 10.1016/j.yebeh.2014.04.022 24840753

[22] Hutto B, Syphilis in Clinical Psychiatry: A review. Psychosomatics. 2001; 42:453–460. doi: 10.1176/appi.psy.42.6.453 11815679 10.1176/appi.psy.42.6.453

[23] Merriam AE, Aronson MK, Gaston P, Wey SL, Katz I. The psychiatric symptoms of Alzheimer´s Disease. J Am Geriatric Soc 1988; 36:7–12. 10.1111/j.1532-5415.1988.tb03427.x 3335733

[24] Cooper JK, Mungas D, Weller PG. Relation of cognitive status and abnormal behaviors in Alzheimer´s disease. J Am Geriatric Soc 1990; 38:867–70. 10.1111/j.1532-5415.1990.tb05701.x 2201714

[25] Grimes DA, Schulz KF. An overview of clinical research: the lay of the land. Lancet. 2002; 359: 57–61. doi: 10.1016/S0140-6736(02)07283-5 11809203 10.1016/S0140-6736(02)07283-5

[26] Medical Research Council Cognitive Function and Ageing Study. Survey into health problems of elderly people: a comparison of self-report with proxy information. Int J Epidemiol. 2000; 29: 684–703. 10922346 10.1093/ije/29.4.684

